# Epidermal or Dermal Specific Knockout of PHD-2 Enhances Wound Healing and Minimizes Ischemic Injury

**DOI:** 10.1371/journal.pone.0093373

**Published:** 2014-04-02

**Authors:** Andrew S. Zimmermann, Shane D. Morrison, Michael S. Hu, Shuli Li, Allison Nauta, Michael Sorkin, Nathaniel P. Meyer, Graham G. Walmsley, Zeshaan N. Maan, Denise A. Chan, Geoffrey C. Gurtner, Amato J. Giaccia, Michael T. Longaker

**Affiliations:** 1 Hagey Laboratory for Pediatric Regenerative Medicine, Department of Surgery, Division of Plastic and Reconstructive Surgery, Stanford University School of Medicine, Stanford, California, United States of America; 2 Department of Surgery, John A. Burns School of Medicine, University of Hawai’i, Honolulu, Hawai’i, United States of America; 3 Institute for Stem Cell Biology and Regenerative Medicine, Stanford University, Stanford, California, United States of America; 4 Department of Radiation Oncology, University of California San Francisco, San Francisco, California, United States of America; 5 Department of Radiation Oncology, Stanford University School of Medicine, Stanford, California, United States of America; Université Libre de Bruxelles, Belgium

## Abstract

**Introduction:**

Hypoxia-inducible factor (HIF)-1α, part of the heterodimeric transcription factor that mediates the cellular response to hypoxia, is critical for the expression of multiple angiogenic growth factors, cell motility, and the recruitment of endothelial progenitor cells. Inhibition of the oxygen-dependent negative regulator of HIF-1α, prolyl hydroxylase domain-2 (PHD-2), leads to increased HIF-1α and mimics various cellular and physiological responses to hypoxia. The roles of PHD-2 in the epidermis and dermis have not been clearly defined in wound healing.

**Methods:**

Epidermal and dermal specific PHD-2 knockout (KO) mice were developed in a C57BL/6J (wild type) background by crossing homozygous floxed PHD-2 mice with heterozygous K14-Cre mice and heterozygous Col1A2-Cre-ER mice to get homozygous floxed PHD-2/heterozygous K14-Cre and homozygous floxed PHD-2/heterozygous floxed Col1A2-Cre-ER mice, respectively. Ten to twelve-week-old PHD-2 KO and wild type (WT) mice were subjected to wounding and ischemic pedicle flap model. The amount of healing was grossly quantified with ImageJ software. Western blot and qRT-PCR was run on protein and RNA from primary cells cultured *in* *vitro*.

**Results:**

qRT-PCR demonstrated a significant decrease of PHD-2 in keratinocytes and fibroblasts derived from tissue specific KO mice relative to control mice (*p<0.05). Western blot analysis showed a significant increase in HIF-1α and VEGF protein levels in PHD-2 KO mice relative to control mice (*p<0.05). PHD-2 KO mice showed significantly accelerated wound closure relative to WT (*p<0.05). When ischemia was analyzed at day nine post-surgery in a flap model, the PHD-2 tissue specific knockout mice showed significantly more viable flaps than WT (*p<0.05).

**Conclusions:**

PHD-2 plays a significant role in the rates of wound healing and response to ischemic insult in mice. Further exploration shows PHD-2 KO increases cellular levels of HIF-1α and this increase leads to the transcription of downstream angiogenic factors such as VEGF.

## Introduction

Chronic wounds represent a significant health and financial burden to patients [1]. The underlying medical conditions for these non-healing wounds includes diabetes, peripheral vascular disease, sustained pressure due to prolonged immobility, and radiation-induced soft tissue injuries [2]. The increasing incidence and prevalence of chronic wounds has made them a prominent public health concern; slow healing of chronic wounds, such as ulcers, can also lead to infection, sepsis, and eventually amputation. In 2004, an estimated $25 billion was spent on the treatment of 6.5 million chronic wounds in the United States alone [1].

Numerous treatment options for chronic wounds currently exist, including treatment of the underlying pathology (e.g. optimal diabetes care with strict blood glucose control), systemic treatment aimed at improving the local wound environment (e.g. nutritional supplements, pentoxifylline, aspirin, flavonoids, thromboxane alpha-2 agonists, sulodexide) and local treatment aimed at improving the wound environment (e.g. dressings, negative local pressure, pressure-relieving mattresses, application of growth factors, skin-grafting) [2]–[4]. The wide array of treatment modalities currently used in the treatment of chronic wounds belies the deficiency of each individual approach to provide an optimal solution. Left untreated, these wounds can lead to serious morbidity and mortality [3], [5]. The dramatically high costs of chronic wounds, both in terms of financial burden and quality of life, underscores the need for investigating their etiology and developing novel therapeutics based on molecular players involved in the wound healing process.

The molecular pathogenesis of many chronic wounds is still unknown. However, impaired response to tissue hypoxia is believed to be a major factor responsible for diminished wound healing. Hypoxia induces numerous cytokines in the wound microenvironment, including vascular endothelial growth factor (VEGF), transforming growth factor-β (TGF-β), and platelet-derived growth factor (PDGF) [6]. Hypoxia-inducible factor-1 (HIF-1), a heterodimeric transcription factor complex consisting of a hypoxia-stabilized α-subunit (HIF-1α) and a constitutively expressed β-subunit (HIF-1β), mediates the cellular response to hypoxia. Under hypoxic conditions, HIF-1α translocates to the nucleus and dimerizes with HIF-1β, allowing binding of the complex to the hypoxia response element (HRE) present in the regulatory sequences of a number of genes vital to cell survival [7]. As a result, HIF-1α is critical for the expression of multiple angiogenic growth factors, cell motility, and the recruitment of endothelial progenitor cells [8]–[10]. HIF-1α expression is induced in normal wound healing [11], [12], while chronic wounds contain low levels of HIF-1α [13], [14]. In light of this evidence linking HIF-1α to wound healing, strategies to manipulate its expression and function are needed.

Prolyl hydroxylase domain-2 (PHD-2) is an oxygen-dependent negative regulator of HIF-1α protein stability [15]. Inhibition of PHD-2 leads to various cellular and physiological responses to hypoxia including: HIF-1α stabilization, induction of hypoxia-inducible genes, stimulation of angiogenesis, and protection against metabolic stress [16], [17]. Recent data from our group showed that PHD-2 also plays an important role in the angiogenic switch independent of HIF-1α stability [18]. In aged wound models, PHD-2 is unregulated and increased, further supporting a role in wound healing [19]. The role of PHD-2 in tissue repair remains unknown. Tissue specific knockout of PHD-2 allows for HIF-1α stabilization and enables a more in-depth study of the role of this master hypoxia regulator within the skin epidermis and dermis.

Since chronic wounds extend through the full thickness of the integument, this study focuses more specifically on the epidermal and dermal layers. Looking at both compartments individually would allow further therapeutic techniques to directly target the compartment most effective at accelerating rates of wound closure. The aim of this study is to utilize a genetic approach to abrogate PHD-2 function in tissue specific compartments and to determine whether the inhibition of HIF-1α degradation would stimulate angiogenesis and accelerate healing.

## Methods

### Ethics

All mouse experiments were performed under the guidance of the Stanford University Veterinary Department and approved by the Institutional Animal Care and Use Committee of Stanford University (protocol number: 21308).

### Creation of PHD-2 Knockout Mouse Model

Mice heterozygously expressing the Cre recombinase under human Keratin 14 promoter (K14-Cre) (Stock #004782) [20] and Collagen 1α2 (Col1α2-Cre-ER) (Stock #016237) [21] as well as mice that were homozygous for loxP sites on either side of exon 2 and 3 in Engl1 (floxed PHD-2) (Stock #009672) were purchased from The Jackson Laboratory (Bar Harbor, Maine) and cross bred until the progeny were heterozygous K14-Cre/homozygous floxed PHD-2 and heterozygous Col1α2-Cre-ER/homozygous floxed PHD-2. The knockout of PHD-2 was constitutive in the K14 mice and tamoxifen-inducible in the Col1α2 mice. The mice that were tamoxifen-inducible were injected with tamoxifen over a period of 5 days before experiments were performed or tissues were harvested. All mice were viable and bred to form a colony. The mice were genotyped using the primers listed in **Table 1**. C57BL/6J mice (Stock #000664) were purchased from The Jackson Laboratory as controls.

**Table 1:** 

	Forward Primer Sequences	Reverse Primer Sequences
PHD-2	CATGTCACGCATCTTCCATC	GATAAACGGCCGAACGAAA
HIF-1	AAACTTCAGACTCTTTGCTTCG	CGGCGAGAACGAGAAGAA
VEGF	AATGCTTTCTCCGCTCTGAA	GCTTCCTACAGCACAGCAGA
B-Actin	GGCTGTATTCCCCTCCATCG	CCAGTTGGTAACAATGCCATGT

### Primary Keratinocyte Harvest

Primary keratinocytes were harvested from the tails of adult (12–15 weeks) C57BL/6J and heterozygous K14-Cre/homozygous floxed PHD-2 mice as described previously [22]. Tails were used as a donor site to minimize the amount of hair follicle cell contamination. The skin (both epidermis and dermis) was removed from the tail and floated on Trypsin without EDTA (Cellgro, Mediatech, Manassas, Virginia) overnight at 4°C to separate the dermis from the epidermis. Following the removal of the dermis, the epidermis was minced and triturated with Serum-Free Keratinocyte Medium (KGM-2 w/o Ca^++^ BulletKit, Lonza, Basel, Switzerland) and filtered through 0.45 μm filter (BD Falcon, BD Biosciences, Bedford, Massachusetts). The filtrate was centrifuged at 1,000 rpm for 5 minutes at 4°C. After the medium was aspirated and replaced, the cells were plated in one well of a six well plate coated with 1 mL fibronectin/collagen coating matrix (Gibco, Life Technologies, Grand Island, New York). Cells were supplemented with keratinocyte medium containing several growth factors and antibiotics (KGM-2 BulletKit plus growth supplements, Lonza). After incubating for 72 hours at 37°C, 5% CO_2_, the medium was aspirated along with non-adherent cells and debris, then washed with PBS (pH 7.4, Gibco) and fresh medium was added. Medium was changed every day until cells were confluent and ready to be used in experiments. All experiments used passage zero cells.

### Primary Fibroblast Harvest

Primary fibroblasts were harvested from the dorsal and ventral skin of adult C57BL/6J and heterozygous Col1α2-Cre-ER/homozygous floxed PHD-2 mice as shown previously [22]. Briefly, 12–15 week old mice were euthanized, shaved and depilated. The skin was cleansed with alcohol pads (PDI, Orangeburg, New York), betadine (Purdue Pharma, Stamford, Connecticut) and once again with alcohol pads. The tissue was harvested and washed in a series of betadine dilutions in PBS (100% betadine, 10% betadine and 100% PBS) then placed in a 50 mL conical tube containing PBS with 100 ug/mL penicillin, 100 ug/mL streptomycin, 50 ug/mL Fungizone, and 40 ug/mL Gentamycin (Thermo Fisher Scientific, Waltham, Massachusetts) on ice. The tissue was then minced, and incubated in Liberase (Roche, Basel, Switzerland) for 1 hour at 37°C with constant shaking at 150 rpm. The primary fibroblasts were cultured in Dulbecco’s Modified Eagle Medium+sodium pyruvate (110 mg/L) (Gibco) supplemented with 10% fetal bovine serum (Axenia BioLogix, Dixon, California), 100 U/mL penicillin, 100 ug/mL streptomycin, 50 ug/mL Fungizone, and 40 ug/mL Gentamycin. After incubating for 48 hours at 37°C, 5% CO_2_, the media and nonadherent cells were aspirated and fresh medium was added. Medium was changed every 2 days until the cells were ready for experiments. All experiments were performed with cells at passage zero or one.

### Western Blot Analysis

Primary fibroblasts and keratinocytes from C57BL/6J, heterozygous K14-Cre/homozygous floxed PHD-2, and heterozygous Col1α2-Cre-ER/homozygous floxed PHD-2 mice were harvested in PBS (pH 7.4) and centrifuged at 1000 rpm for 5 minutes at 4°C. The cell pellets were then lysed in Urea Lysis Buffer (9 M urea, 75 mM Tris [pH 7.5], 150 mM β-mercaptoethanol). Cells were sonicated briefly (10 seconds), placed on ice for 5 minutes, and then sonicated again. Protein concentrations were determined by the Bradford assay (Bio-Rad Laboratories, Hercules, California). Thirty-five μg of protein from each group were resolved on sodium dodecyl sulfate (SDS)-polyacrylamide gels (Invitrogen, Carlsbad, California) and then transferred onto polyvinyl difluoride membranes (Invitrogen). Primary antibodies against mouse HIF-1α (1∶500, Novus Biologicals, Littleton, Colorado), PHD-2 (1∶500, Novus Biologicals), VEGF (1∶500, Santa Cruz Biotechnology, Dallas, Texas) and B-actin (1∶1000, Santa Cruz Biotechnology) were used for detecting the corresponding proteins. Western blots were developed using Enhanced Chemiluminescence (ECL) Plus (GE Healthcare, Little Chalfont, United Kingdom) and then analyzed using ImageJ software (NIH, Bethesda, Maryland). Blots were run in triplicate from C57BL/6J and heterozygous Col1α2-Cre-ER/homozygous floxed PHD-2 fibroblasts and C57BL/6J and heterozygous K14-Cre/homozygous floxed PHD-2 keratinocytes.

### Quantitative Reverse Transcriptase-Polymerase Chain Reaction Analysis

RNA from fibroblasts and keratinocytes of C57BL/6J, heterozygous K14-Cre/homozygous floxed PHD-2, and heterozygous Col1α2-Cre-ER/homozygous floxed PHD-2 mice was extracted using TRIzol (1 mL per well, Ambion, Life Technologies) and RNeasy Plus Mini Kit (Qiagen, Venlo, Limburg, Netherlands). The RNA was reverse transcribed using the TaqMan Reverse Transcription Reagents (Applied Biosystems, Life Technologies, Grand Island, New York). Quantitative real-time quantitative PCR (qRT-PCR) was run on the ABI Prism 7900HT Sequence Detection System (Applied Biosystems) using Power SYBR Green Master Mix (Applied Biosystems). PCR was performed by denaturing at 95°C for 15 minutes, followed by 40 cycles of denaturation at 95°C for 30 seconds and annealing at 60°C for 1 minute. All gene expression data were normalized to β-Actin. All reactions were run in triplicate. (Primer sequences can be found in **Table 1**).

### Excisional Wound Model

The C57BL/6J, heterozygous K14-Cre/homozygous floxed PHD-2, and heterozygous Col1α2-Cre-ER/homozygous floxed PHD-2 mice were housed in a 12-hour light/dark cycle and provided ad libitum with standard food and water. Female mice ages 10–12 weeks were prepped for surgery (n = 3). After depilation, two 6 mm full-thickness excisional wounds extending through the panniculus carnosus were made on dorsa of mice as previously described [23]. A donut-shaped 12 mm silicone splint (Invitrogen) was placed and fixed to the skin with cyanoacrylate glue and interrupted 6-0 nylon sutures so that the wound was centered within the splint. A Tegaderm (3 M, St. Paul, Minnesota) dressing was placed over the wounds and the animals were housed individually. Digital imaging, as well as redressing of the wounds, was preformed every other day until closure. Time to wound closure was defined as the time at which the wound bed was completely re-epithelialized and filled with new tissue. Wound area was quantified by tracing wound margins and calculated as a percent of the original wound size using ImageJ software, with scaling normalized to the circular reference of the splint.

### Ischemic Pedicle Flap Model

Ten to twelve week old male mice, with C57BL/6J, heterozygous K14-Cre/homozygous floxed PHD-2, and heterozygous Col1α2-Cre-ER/homozygous floxed PHD-2 mice were subjected to a standardized, reproducible *in vivo* model of soft tissue ischemia (n = 3) as shown previously [24]. This was achieved by creating a U-shaped peninsular incision (1.25 cm in width and 2.5 cm in length) on the dorsal side of mice penetrating the epidermis, dermis, and underlying adipose tissue. This tissue was elevated from the underlying muscle bed and a 0.13 mm thick silicone sheet (Invotec International, Jacksonville, Florida) was inserted to separate the skin from the underlying tissue bed before the flap was sutured back in place. The silicone sheet blocks blood supply from the underlying wound bed and forces revascularization to come from the cranial axial blood supply. The flap was sutured using interrupted 6-0 sutures along all three sides of the flap. The flap was covered with Tegaderm and the mice were housed individually. Digital images of the flaps were taken on day 0 and 9. The degree of ischemia within the tissue was confirmed using ImageJ software where the area of ischemia was measured and divided by the overall flap area to determine the percent of ischemia.

### Immunofluorescence

After fixation in 4% paraformaldehyde, tissue samples were embedded in paraffin and sliced into sections 8 μm in thickness using a microtome. PHD-2 expression was assessed by incubating slides overnight at 4°C using a polyclonal rabbit anti-mouse anti-PHD-2 primary antibody (1∶100, #100–2219, Novus Biologicals) with secondary staining using Alexa Fluor 594 Goat Anti-Rabbit IgG (1∶200, Invitrogen) at room temperature for 1 hour. HIF-1α expression was assessed by incubating slides overnight at 4°C using a polyclonal rabbit anti-mouse anti-HIF-1α primary antibody (1∶100, #100–479, Novus Biologicals) with secondary staining using Alexa Fluor 594 Goat Anti-Rabbit IgG (1∶200, Invitrogen) at room temperature for 1 hour. VEGF expression was assessed by incubating slides overnight at 4°C using a polyclonal rabbit anti-mouse anti-VEGF primary antibody (1∶100, #46154, Abcam, Cambridge, UK) with secondary staining using Alexa Fluor 594 Goat Anti-Rabbit IgG (1∶200, Invitrogen) at room temperature for 1 hour. Neovascularization was assessed by incubating slides overnight at 4°C using a polyclonal rabbit anti-mouse anti-CD31 primary antibody (1∶100, #28364, Abcam) with secondary staining using Alexa Fluor 594 Goat Anti-Rabbit IgG (1∶200, Invitrogen) at room temperature for 1 hour. All samples were counterstained with DAPI. Slides were mounted with VECTASHIELD Mounting Medium (Vector Laboratories, Burlingame, California) and cover-slipped. A Zeiss Axioplan 2 fluorescence microscope was used to image the slides (Carl Zeiss, Thornwood, New York). Quantification of fluorescence was performed by a blinded observer using ImageJ software and depicted as percent of relative expression.

### Statistical Analysis

Data were analyzed by two-tailed student’s *t* test and presented as mean ± standard error. A *p* value <0.05 was considered statistically significant.

## Results

### qRT-PCR Assessment of PHD-2 Knockout

**Figure 1:**
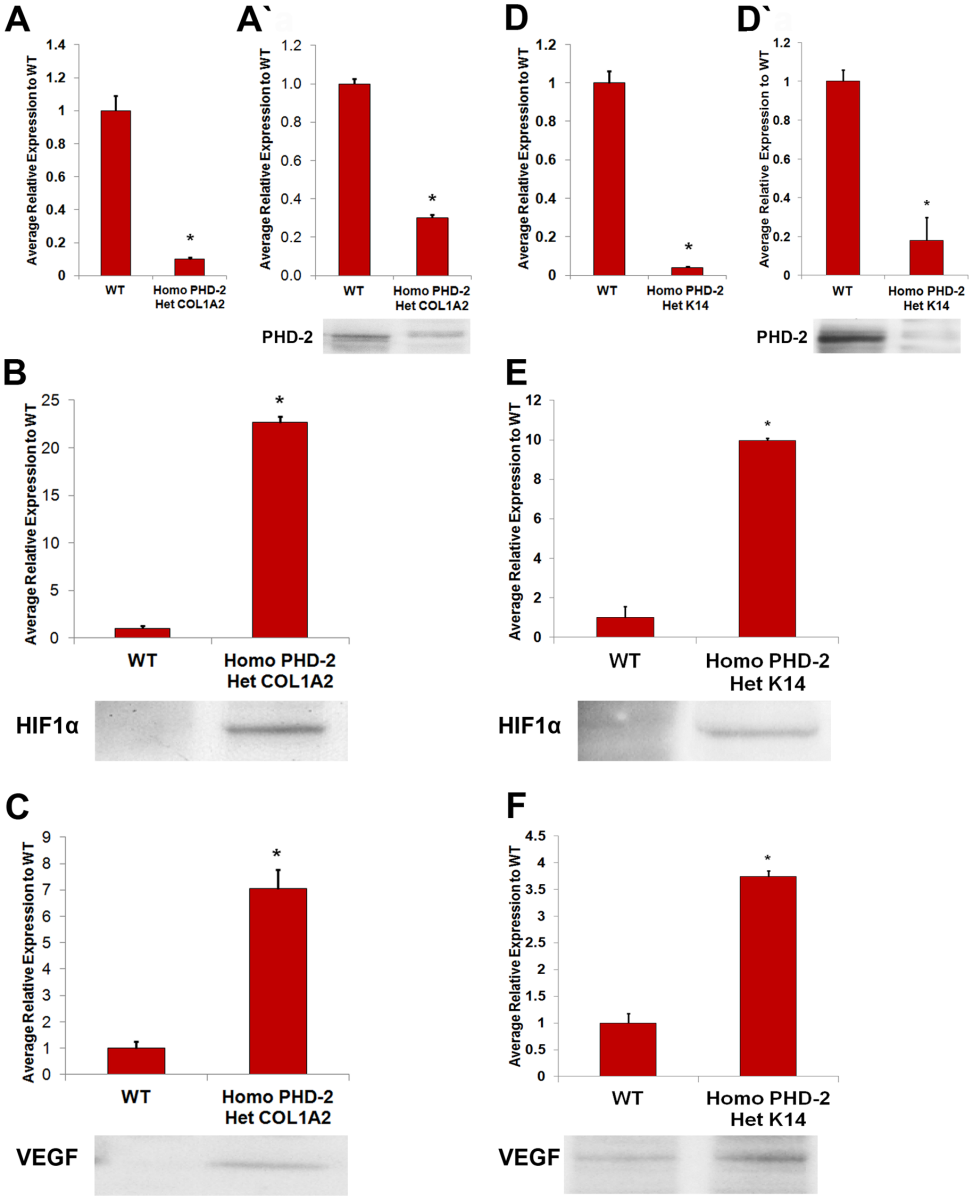
*In vitro* analysis of PHD-2 knockout and protein quantification. **A**) Quantitative polymerase chain reaction for PHD-2 knockout in heterozygous Col1α2-Cre-ER/homozygous floxed PHD-2 fibroblasts compared to wild type fibroblasts (*p<0.05). **A’**) Western blot data for PHD-2 knockout in fibroblasts of heterozygous Col1α2-Cre-ER/homozygous floxed PHD-2 mice compared to wild type mice (*p<0.05). **B**) Western blot data for HIF-1α in fibroblasts of heterozygous Col1α2-Cre-ER/homozygous floxed PHD-2 mice compared to wild type mice (*p<0.05). **C**) Western blot data for VEGF in fibroblasts of heterozygous Col1α2-Cre-ER/homozygous floxed PHD-2 mice compared to wild type mice (*p<0.05). **D**) Quantitative polymerase chain reaction for PHD-2 knockout in heterozygous K14-Cre/homozygous floxed PHD-2 keratinocytes compared to wild type keratinocytes (*p<0.05). **D’**) Western blot data for PHD-2 knockout in keratinocytes of heterozygous K14-Cre/homozygous floxed PHD-2 mice compared to wild type mice (*p<0.05). **E**) Western blot data for HIF-1α in keratinocytes of heterozygous K14-Cre/homozygous floxed PHD-2 mice compared to wild type mice (*p<0.05). **F**) Western blot data for VEGF in keratinocytes of heterozygous K14-Cre/homozygous floxed PHD-2 mice compared to wild type mice (*p<0.05).

qRT-PCR was used to assess the knockout of PHD-2 at the mRNA level. In theory, transcription of PHD-2 should be severely depressed and that of HIF-1α mRNA should not be altered since PHD-2 regulates HIF-1α at the protein level. As expected, mRNA levels of PHD-2 were drastically reduced in keratinocytes of heterozygous K14-Cre/homozygous floxed PHD-2 mice and in the fibroblasts of heterozygous Col1a2-Cre-ER/homozygous floxed PHD-2 mice. In contrast, PHD-2 mRNA was unaltered in the control animals (*p<0.05) (**Figure 1A and D**).

### Western Blot Analysis

To determine if PHD-2 was being efficiently knocked down in the epidermis and dermis, primary keratinocytes and fibroblasts from C57BL/6J, heterozygous K14-Cre/homozygous floxed PHD-2, and heterozygous Col1α2-Cre-ER/homozygous floxed PHD-2 were run on western blots for PHD-2, HIF-1α, and VEGF (**Figure 1**). Fibroblasts from heterozygous Col1α2-Cre-ER/homozygous floxed PHD-2 mice showed significantly lower levels of PHD-2 and higher levels of HIF-1α and VEGF than the control mice (*p<0.05) (**Figure 1A’–C**). Similarly keratinocytes from the heterozygous K14-Cre/homozygous floxed PHD-2 mice showed significantly lower levels of PHD-2 and increased levels of HIF-1α and VEGF compared to control (*p<0.05) (**Figure 1D’–F**).

### Wound Closure and Necrosis

**Figure 2:**
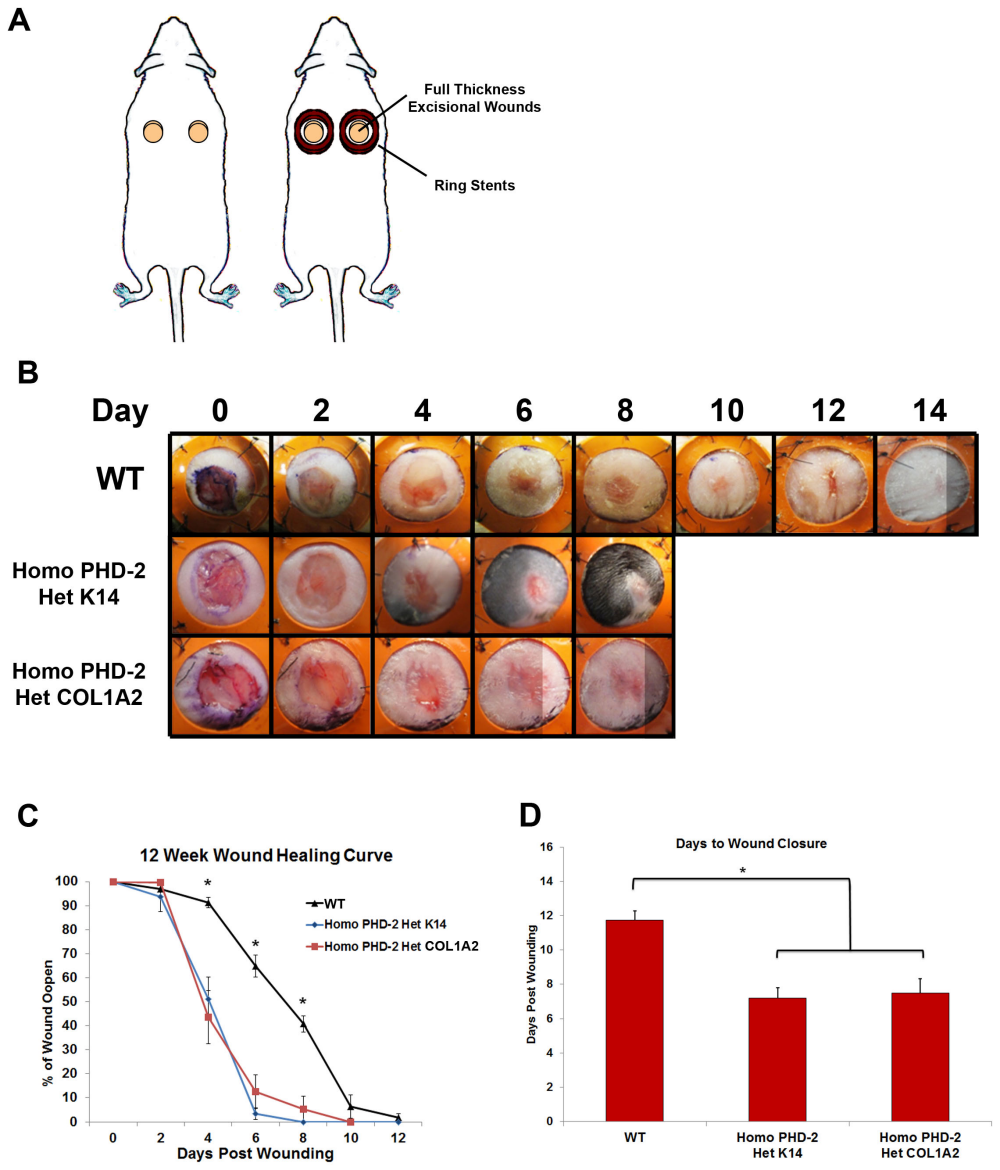
*In vivo* data from humanized wound healing model. **A**) Schematic of stented full-thickness excisional wound model. **B**) Representative pictures of wounds from days 0–14 for wild type, heterozygous K14-Cre/homozygous floxed PHD-2, and heterozygous Col1α2-Cre-ER/homozygous floxed PHD-2 mice. **C**) Twelve week wound healing curve showing the wound closure rate for wild type, heterozygous K14-Cre/homozygous floxed PHD-2, and heterozygous Col1α2-Cre-ER/homozygous floxed PHD-2 mice with significant differences on days 4, 6, and 8 (*p<0.05). **D**) Graph representing days to wound closure with a significant difference in days to closure between the wild type and the heterozygous K14-Cre/homozygous floxed PHD-2 and heterozygous Col1α2-Cre-ER/homozygous floxed PHD-2 mice (*p<0.05).

A humanized wound healing model that heals through granulation tissue and re-epithelialization was used to test *in vivo* rates of wound closure (**Figure 2A**). There was a significant difference in the rates of wound closure across all mice tested (**Figure 2B and C**). The wounds on the mice from the control group (C57BL/6J) closed on day 11.75±1.67 whereas the transgenic mice (heterozygous K14-Cre/homozygous floxed PHD-2 and heterozygous Col1α2-Cre-ER/homozygous floxed PHD-2) healed on post-injury day 7.2±1.03 and 7.5±1.2 (*p<0.05) (**Figure 2C**). While the first 2 days did not show a statistical significance between the control groups and the PHD-2 knockout mice, there was a significant difference on days 4, 6, and 8. In fact, the wounds were completely re-epithelialized in the knockout mice by day 8. The most striking difference in rates of wound closure occurred at the later stages of wound healing (**Figure 2D**). There was no difference between the healing rate between the epidermal and dermal **PHD-2 knockout mice.**

**Figure 3:**
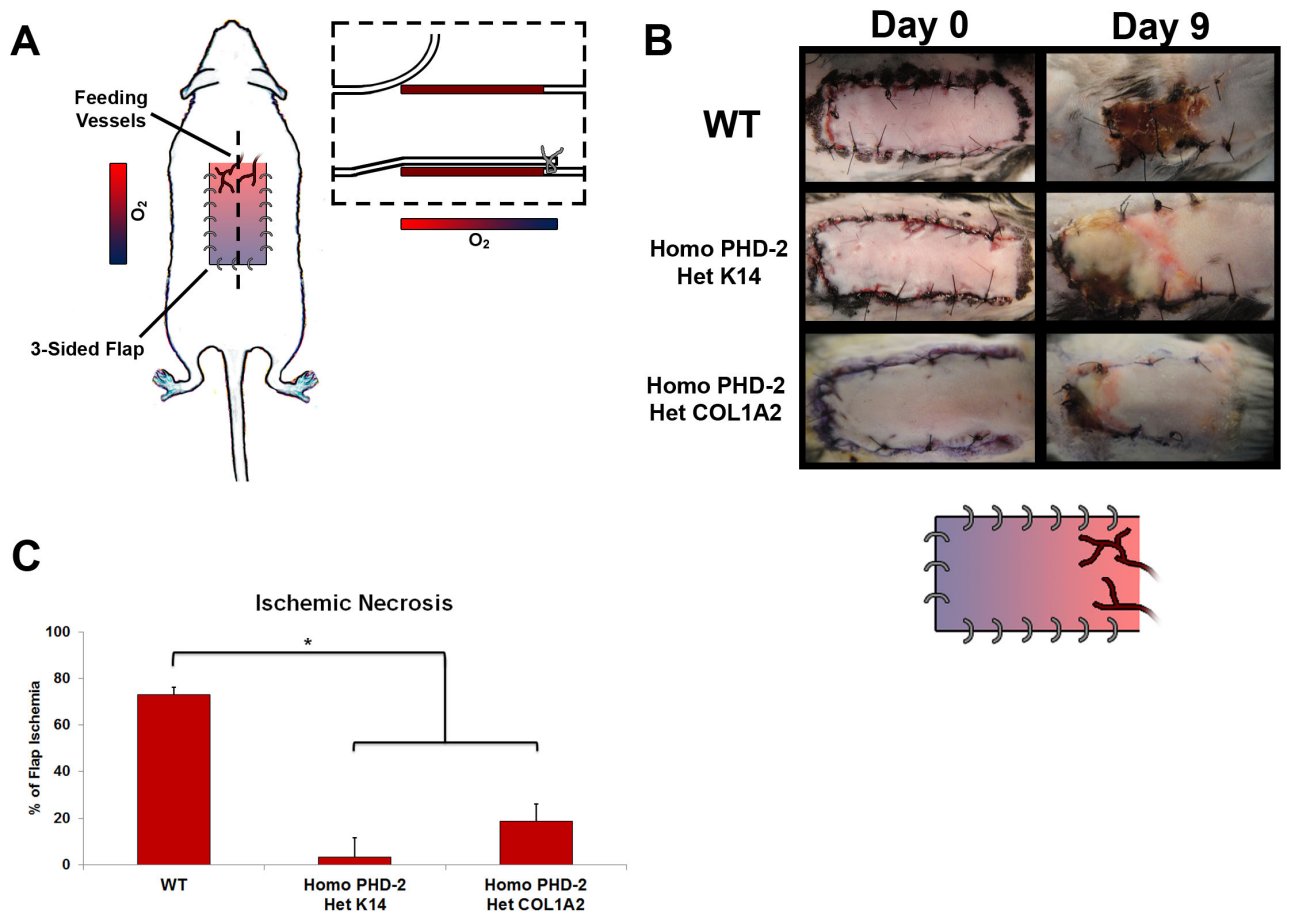
*In vivo* data from ischemic pedicle flap model. **A**) Schematic of ischemic pedicle flap model. **B**) Representative figures taken at day 0 and 9 of ischemic flaps for wild type, heterozygous K14-Cre/homozygous floxed PHD-2, and heterozygous Col1α2-Cre-ER/homozygous floxed PHD-2 mice. A schematic of tissue reperfusion gradient is also shown. **C**) Graphical representation of total flap ischemia with a significant difference (*p<0.05) between the wild type and the heterozygous K14-Cre/homozygous floxed PHD-2, and heterozygous Col1α2-Cre-ER/homozygous floxed PHD-2 mice.

Continuing to investigate the effects of PHD-2 knockout *in vivo*, the ischemic pedicle flap was used to look at tissue perfusion (**Figure 3A**). This is a model that investigates blood supply and tissue perfusion. There was significantly less ischemia and necrosis in the PHD-2 knockout mice when compared to the control groups (**Figure 3B and C**). The ischemia in C57BL/6J mice was 73.8±17.9%. In the heterozygous K14-Cre/homozygous floxed PHD-2 mice and the heterozygous Col1α2-Cre-ER/homozygous floxed PHD-2 mice, ischemia was significantly lower at 3.5±6% and 18.71±16.4%, respectively (*p<0.05).

### Histology

**Figure 4:**
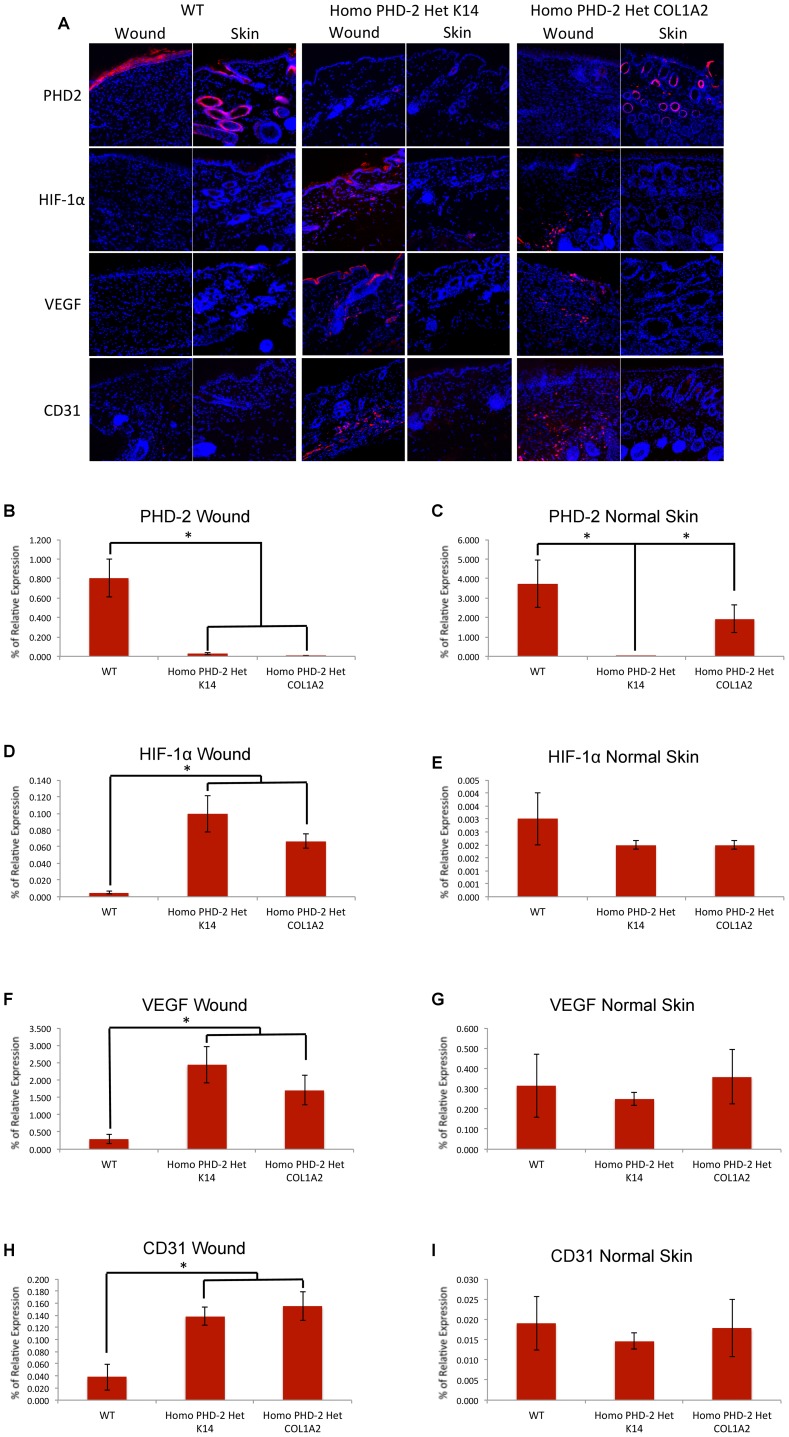
Immunofluorescence of wounded tissue and normal unwounded skin. **A**) Representative images for immunofluorescent staining of PHD-2, HIF-1α, VEGF, and CD31 on healed wounded skin and adjacent normal unwounded skin of wild type, heterozygous K14-Cre/homozygous floxed PHD-2, and heterozygous Col1α2-Cre-ER/homozygous floxed PHD-2 mice at 400× magnification. **B**) Relative protein immunolocalization of PHD-2 in healed wounds of wild type, heterozygous K14-Cre/homozygous floxed PHD-2, and heterozygous Col1α2-Cre-ER/homozygous floxed PHD-2 mice (*p<0.05). **C**) Relative protein immunolocalization of PHD-2 in adjacent normal unwounded skin of wild type, heterozygous K14-Cre/homozygous floxed PHD-2, and heterozygous Col1α2-Cre-ER/homozygous floxed PHD-2 mice (*p<0.05). **D**) Relative protein immunolocalization of HIF-1α in healed wounds of wild type, heterozygous K14-Cre/homozygous floxed PHD-2, and heterozygous Col1α2-Cre-ER/homozygous floxed PHD-2 mice (*p<0.05). **E**) Relative protein immunolocalization of HIF-1α in adjacent normal unwounded skin of wild type, heterozygous K14-Cre/homozygous floxed PHD-2, and heterozygous Col1α2-Cre-ER/homozygous floxed PHD-2 mice. **F**) Relative protein immunolocalization of VEGF in healed wounds of wild type, heterozygous K14-Cre/homozygous floxed PHD-2, and heterozygous Col1α2-Cre-ER/homozygous floxed PHD-2 mice (*p<0.05). **G**) Relative protein immunolocalization of VEGF in adjacent normal unwounded skin of wild type, heterozygous K14-Cre/homozygous floxed PHD-2, and heterozygous Col1α2-Cre-ER/homozygous floxed PHD-2 mice. **H**) Relative immunolocalization of CD31 in healed wounds of wild type, heterozygous K14-Cre/homozygous floxed PHD-2, and heterozygous Col1α2-Cre-ER/homozygous floxed PHD-2 mice (*p<0.05). **I**) Relative immunolocalization of CD31 in adjacent normal unwounded skin of wild type, heterozygous K14-Cre/homozygous floxed PHD-2, and heterozygous Col1α2-Cre-ER/homozygous floxed PHD-2 mice.

Based on the increase of HIF-1 and VEGF *in vitro*, and the accelerated rate of wound closure as well as the minimization of tissue ischemia *in vivo*, we suspected an increased blood supply in the wounds of the knockout mice via upregulation of the HIF pathway. Tissue harvested from full-thickness excisional wounds and adjacent unwounded normal skin was sectioned and stained for PHD-2, HIF-1α, VEGF, and CD31, and imaged at 400× magnification. Representative images are shown in **Figure 4A**. When sections of wounds were analyzed for positive PHD-2 staining, heterozygous K14-Cre/homozygous floxed PHD-2 mice and heterozygous Col1α2-Cre-ER/homozygous floxed PHD-2 mice had significantly decreased PHD-2 immunolocalization per field of view than wild type control mice (*p<0.05) (**Figure 4B**). The downstream effects of PHD-2 knockout were evident by increased HIF-1α and VEGF protein immunolocalization on wounds of heterozygous K14-Cre/homozygous floxed PHD-2 mice and heterozygous Col1α2-Cre-ER/homozygous floxed PHD-2 mice versus wild type controls (*p<0.05) (**Figure 4D and F**). Consequently, there was increased immunofluorescence of CD31 on the wounds of heterozygous K14-Cre/homozygous floxed PHD-2 mice and heterozygous Col1α2-Cre-ER/homozygous floxed PHD-2 mice (**Figure 4H**). Interestingly, PHD-2 immunolocalization was increased in normal unwounded skin of both wild type and heterozygous Col1α2-Cre-ER/homozygous floxed PHD-2 mice (*p<0.05) (**Figure 4C**). However, the immunolocalization of HIF-1α, VEGF, and CD31 in unwounded skin were not significantly different between both PHD-2 knockout and wild type mice (**Figure 4E, G, and I**).

## Discussion

Clinical approaches to the treatment of wounds are numerous, but individually often do not provide an ideal solution. Problems with local administration of vulnerary agents and low efficacy are the main drawbacks [25]. The development of animal models that recapitulate potential genetic changes needed for accelerated wound closure would greatly contribute to the field of chronic wound care research. Moreover, such animal models would allow the screening of novel therapeutics. In this study, the tissue specific PHD-2 knockout mouse provides a good example of such a model. The present study has shown that epidermal and dermal specific knockout of PHD-2 accelerates the rate of excisional wound closure, as well as increasing flap survival in an ischemic pedicle flap model. These results confirm the importance of PHD-2 as the negative regulator of HIF-1α, and of HIF-1α being an important part of the wound healing process not only in wound closure but also by improving local tissue perfusion as demonstrated by increased flap survival. Our data also provide a benchmark of efficacy against which novel therapeutic modalities could be compared.

The results of the present study confirm and elaborate on previous studies that point to PHD-2 as a negative regulator of wound closure [26]–[28]. To date, the exact role of PHD-2 in the epidermis and dermis along with its relation to wound closure has not been explored. By forming a deeper understanding of the role that these proteins and transcription factors play in wound healing and homeostasis of local oxygen tension and tissue perfusion, it may be possible to more effectively guide the development of clinical therapeutics for the treatment of chronic wounds and improvement of flap survival. Furthermore, understanding PHD-2 and HIF-1 under inhibited and normal states can lead to a better understanding of their function in diseased states such as cancer where angiogenic factors are over-expressed and can lead to the growth and metastasis of cells [18].

Our mouse models provide a platform to gain a better understanding of the genetic implications of PHD-2 and HIF-1 in wound healing. However, further studies on the safety and efficacy of knocking out PHD-2 are required before such therapeutic regimens can be moved from bench to bedside, perhaps with a clinically safe local delivery of “knockout” strategies to PHD-2. While concerns exist regarding the possibility of promoting tumorigenesis and metastasis by enhancing angiogenesis through inhibition of a negative regulator of a proangiogenic transcription factor such as HIF-1, it is worth noting that no obvious effects of tumorigenesis have been noticed in mice that have been kept for up to 1.5 years.

Elucidating the gene regulatory network, which governs angiogenesis in the context of chronic wounds is clearly a worthwhile cause. Further study of the role of PHD-2, HIF-1, and their different isoforms is therefore needed in order to advance our understanding of this network and identify possible targets for pharmacological modulation.
